# Leveraging plant-derived nanovesicles for advanced nucleic acid-based gene therapy

**DOI:** 10.7150/thno.104507

**Published:** 2025-01-01

**Authors:** Meihong Chai, Bowen Gao, Shihua Wang, Liping Zhang, Xing Pei, Baosen Yue, Xueyan Zhen, Mingzhen Zhang

**Affiliations:** 1 Xi'an Hospital of Traditional Chinese Medicine, Xi'an, Shaanxi, 710021, China.; 2 Xi'an Jiaotong University Health Science Center, Xi'an, Shaanxi, 710061, China.; 3 School of Medicine, Xi'an Peihua University, Xi'an, Shaanxi, 710125, China.; 4 School of Basic Medicine and Forensic Medicine, Henan University of Science and Technology, Luoyang, Henan, 471000, China.; 5 Tianjin Key Laboratory of Food and Biotechnology, School of Biotechnology and Food Science, Tianjin University of Commerce, Tianjin 300134, China.

**Keywords:** Plant-derived nanovesicles, Gene therapy, RNA, DNA, Nanomedicine, Delivery system

## Abstract

Gene therapy has evolved into a pivotal approach for treating genetic disorders, extending beyond traditional methods of directly repairing or replacing defective genes. Recent advancements in nucleic acid-based therapies-including mRNA, miRNA, siRNA, and DNA treatments have expanded the scope of gene therapy to include strategies that modulate protein expression and deliver functional genetic material without altering the genetic sequence itself. This review focuses on the innovative use of plant-derived nanovesicles (PDNVs) as a promising delivery system for these nucleic acids. PDNVs not only enhance the stability and bioavailability of therapeutic nucleic acids but also improve their specificity and efficacy in targeted gene therapy applications. They have shown potential in the treatment of various diseases, including cancer and inflammatory conditions. By examining the unique properties of PDNVs and their role in overcoming the limitations of conventional delivery methods, this review highlights the transformative potential of PDNV-based nucleic acid therapies in advancing the field of gene therapy.

## Introduction

Gene therapy has emerged as a transformative approach to the treatment of genetic disorders, offering the ability to precisely regulate and correct malfunctioning genes within cells 1. Initially focused on directly repairing or replacing defective genes, gene therapy has expanded to include a wider range of nucleic acid-based therapies. These therapies include small interfering RNA (siRNA) 2-4, microRNA (miRNA) 5-7, messenger RNA (mRNA) 8-12, and DNA treatments 13-16, which aim to modulate gene expression or deliver functional genetic material. By focusing on these strategies, gene therapy provides therapeutic effects without necessitating direct alterations to the genetic sequence. The integration of nucleic acid-based therapies with traditional treatment methods allows for a more comprehensive targeting of cellular pathways, enhancing therapeutic outcomes and minimizing side effects. This has generated substantial interest among researchers and clinicians, highlighting the potential of gene therapy to revolutionize disease management.

Despite its promise, the effective delivery of nucleic acids remains a significant challenge. Direct administration of naked nucleic acids can result in non-specific distribution, low cellular uptake, rapid clearance, and susceptibility to enzymatic degradation 17-20. To address these challenges, various delivery methods have been developed. Physical techniques, such as nuclear transfection and electroporation, often face limitations* in vivo* due to potential cellular damage. While viral vectors can facilitate efficient gene transfection, they are associated with issues such as immunogenicity, biosafety concerns, complex preparation, limited packaging capacity, and poor targeting specificity 21, 22. Compared with traditional methods, non-viral vectors, such as cationic polymers 23-25, cationic liposomes 26, 27, and nanoparticles 28-30, offer the advantages of easier preparation, lower toxicity, and higher gene packaging capacity. Among these, plant-derived nanovesicles (PDNVs), specifically, are nanoscale vesicles secreted by plant cells that contain DNA, small RNAs (sRNAs), miRNAs, and proteins. These vesicles mediate intercellular communication and exert effects such as anti-inflammatory, antiviral, antifibrotic, and antitumor activities, contributing to the plant's defense mechanisms against pathogens. Structurally, they exhibit a lipid bilayer that encapsulates various bioactive molecules. This unique structure enables them to carry and transport diverse bioactive substances. Additionally, most PDNVs are edible and can serve as carriers for targeted drug delivery without toxicity or side effects, making them an emerging focus in research. Therefore, PDNVs have shown promise as carriers for delivering therapeutic molecules due to their unique properties that enhance their biocompatibility and minimize adverse immune responses 31, making them safer for therapeutic applications.

**Figure 1:**
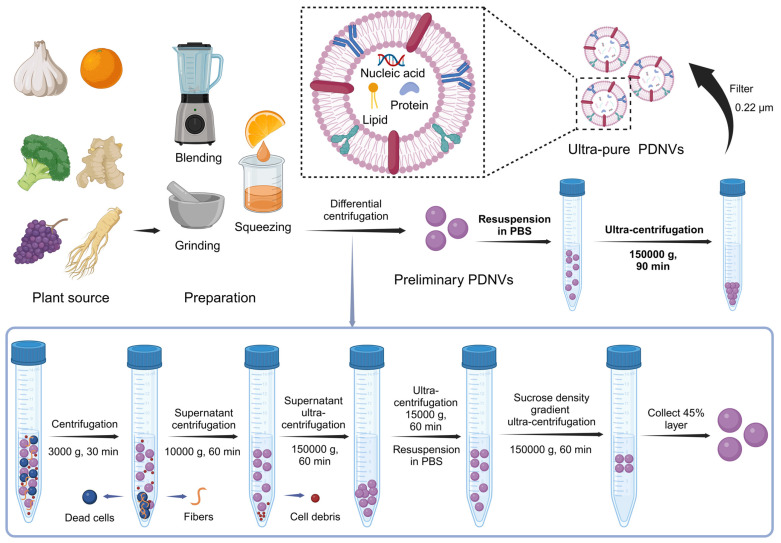
** Isolation and Preparation of PDNVs.** Plant materials such as garlic, broccoli, ginger, grape, and ginseng are used as sources for PDNVs. The extraction process involves blending, grinding, and squeezing to obtain plant juice. The resulting extract undergoes a series of centrifugation steps to remove dead cells, fibers, and cellular debris. Initial centrifugation at 3000 g for 30 min sediments dead cells and fibers at the bottom of the tube, allowing the collection of the supernatant for further processing. The supernatant is then subjected to 10,000 g centrifugation for 60 min to remove additional debris, followed by ultracentrifugation at 150,000 g for 60 min to pellet PDNVs. The pellet is resuspended in PBS and further purified using a sucrose density gradient ultracentrifugation (150,000 g for 60 min) to isolate the 45% layer containing purified PDNVs, which are finally washed and filtered through a 0.22 µm membrane to obtain ultra-pure PDNVs containing nucleic acids, proteins, and lipids (created with Biorender.com).

In addition to mammalian cells, plant cells also produce multivesicular bodies (MVBs) through interactions between the plasma membrane and cell wall, leading to the formation of paravesicles. The presence of MVBs in plant cells was first identified in 1967 using transmission electron microscopy, which revealed that carrot cell cultures generate MVBs that fuse with the plasma membrane, releasing membrane-structured vesicles into the extracellular space 32. Since then, PDNVs have been isolated from a range of plants, including ginger 33, 34, grapefruit 35, 36, orange 37, blueberries 38, and various medicinal herbs 39, 40, expanding their application and significance in scientific research. The preparation of PDNVs involves multiple steps to ensure purity and functionality, as depicted in the accompanying **Figure 1**. Garlic, orange, broccoli, ginger, grapes, and ginseng are processed through blending, grinding, or squeezing to obtain a plant extract. This extract undergoes a series of differential centrifugation steps to remove dead cells, fibers, and cell debris. Initial centrifugation at 3000 g for 30 minutes is followed by subsequent centrifugations at 10,000 g and 150,000 g to separate and purify the PDNVs. Further purification is achieved using ultra-centrifugation and sucrose density gradient centrifugation, resulting in ultra-pure PDNVs with minimal contaminants. This process ensures that the final PDNV product is highly enriched in nucleic acids, proteins, and lipids, making them suitable carriers for therapeutic siRNA delivery (**Figure 1**).

Compared to traditional nanocarriers such as lipid nanoparticles (LNPs) and exosomes, PDNVs present unique properties that enhance their potential as therapeutic delivery systems. LNPs, while effective in encapsulating and delivering nucleic acids, often require complex synthetic modifications and may exhibit limited stability *in vivo* due to rapid clearance by the immune system. Exosomes, derived from mammalian cells, show enhanced biocompatibility and targeting capabilities but are constrained by low yield and scalability challenges, as well as potential safety concerns related to their cellular origin. PDNVs, on the other hand, offer notable advantages over synthetic carriers and mammalian exosomes due to their non-toxic, low immunogenic, highly biocompatible properties 41-43, high yield, and low production cost, making them an economically viable option. Therefore, PDNVs offer significant advantages as emerging delivery systems. Firstly, unlike many synthetic and mammalian-derived systems, PDNVs are composed of biomolecules similar to dietary components and are more readily recognized by the body as food-derived, which minimizes immune activation compared to synthetic or animal-derived carriers. Their natural composition aligns with evolutionary tolerance mechanisms in mammals, reducing immune reactivity. Thereby PDNVs exhibit inherent potential for oral delivery due to their stability in the gastrointestinal environment, making them a promising candidate for non-invasive therapies. Inside the body, PDNVs may use distinctive surface markers from their plant origins to target specific cells or tissues. For instance, their behavior in the gastrointestinal tract differs from that of other delivery vectors. This may be due to specific recognition mechanisms with intestinal cells, making PDNVs more likely to exert local effects in the gut or cross the intestinal barrier to enter the bloodstream. Secondly, PDNVs contain therapeutic potential due to their endogenous nucleic acid cargo, which may include functional miRNAs and other small RNAs native to the source plant. This endogenous cargo not only adds an intrinsic therapeutic benefit but may also reduce the need for additional loading processes. Furthermore, PDNVs can encapsulate and protect nucleic acids more effectively, ensuring higher stability and bioavailability 44. The urgency for utilizing PDNVs is underscored by the increasing demand for innovative and efficient delivery systems that can address the limitations of traditional methods, ultimately paving the way for more effective gene therapies 45, 46. These unique characteristics position PDNVs as an innovative and versatile platform in nucleic acid-based gene therapy.

**Figure 2:**
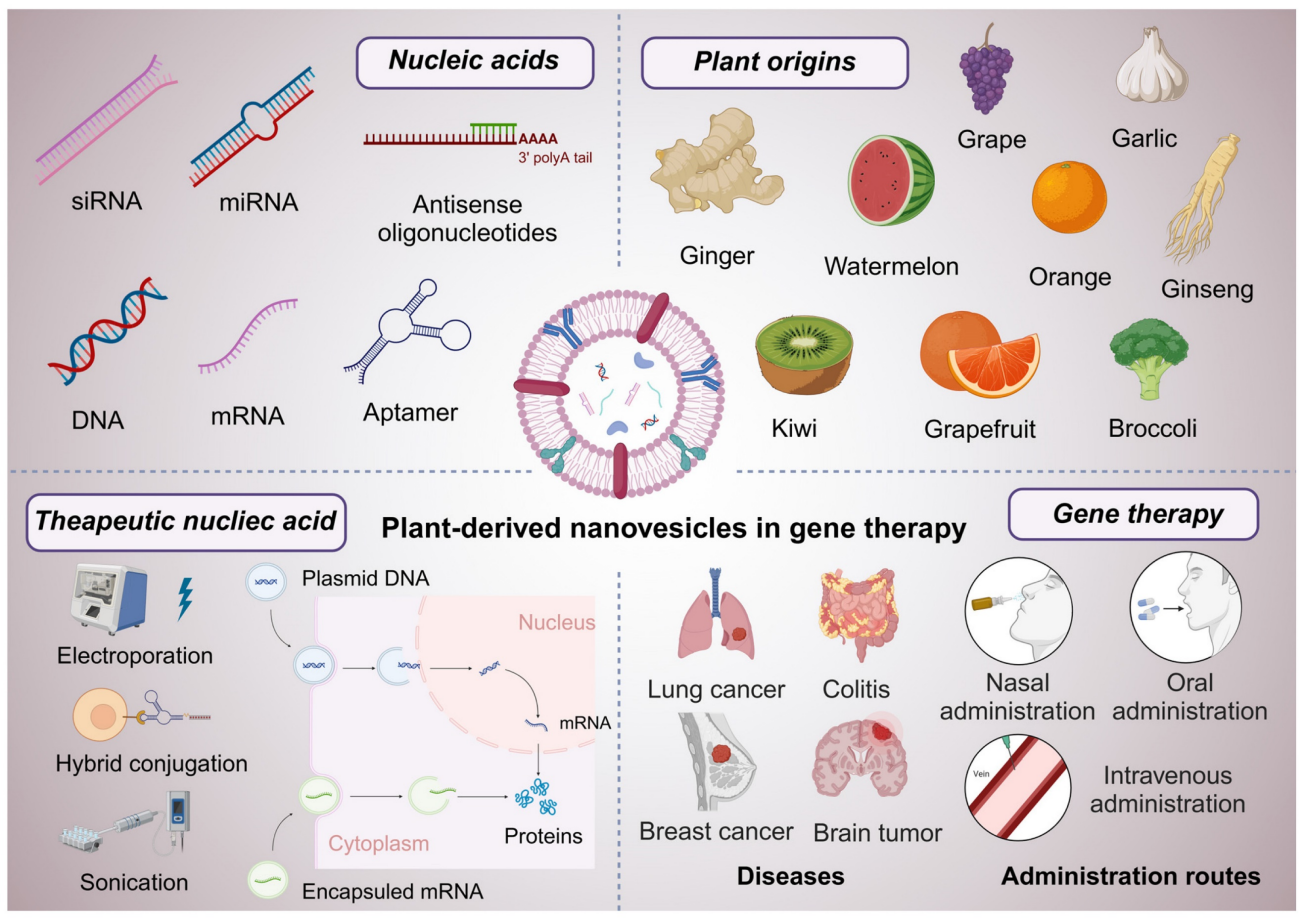
** Graphical abstract of PDNVs in gene therapy.** This scheme illustrates the diverse applications and components of PDNVs in gene therapy. The top sections showcase various nucleic acids (siRNA, miRNA, antisense oligonucleotides, DNA, mRNA, and aptamers) that can be loaded into PDNVs for therapeutic purposes, as well as plant origins (e.g., grape, garlic, ginger, watermelon, orange, ginseng, kiwi, grapefruit, and broccoli) from which PDNVs can be derived. The bottom left panel highlights therapeutic nucleic acid loading strategies for PDNVs, including electroporation, hybrid conjugation, and sonication, and shows how these methods enable nucleic acids to reach target cells and influence gene expression. The bottom center panel depicts diseases that can be treated by PDNV-based therapies, including lung cancer, colitis, breast cancer, and brain tumors. The bottom right section outlines administration routes for PDNVs, such as nasal, oral, and intravenous administration, which facilitate targeted delivery and therapeutic action. This review aims to discuss the emerging trends, therapeutic potential, and application strategies of PDNVs in gene therapy (created with Biorender.com).

This review aims to provide a comprehensive overview of PDNVs and their application as effective carriers for nucleic acid-based therapies in gene therapy (**Figure 2**). We will explore the unique properties of PDNVs, discuss the mechanisms by which they facilitate nucleic acid delivery, and highlight the challenges and opportunities associated with their use. By synthesizing current knowledge in this area, we aim to underscore the transformative potential of PDNV-based therapies in advancing the field of gene therapy.

## PDNVs-based therapeutic siRNA in disease therapy

### The development of therapeutic siRNA

RNA interference (RNAi) is a highly conserved biological process that allows cells to defend against foreign genetic elements by silencing specific genes 47-49. siRNA, a critical component of RNAi, has emerged as a promising therapeutic tool due to its ability to selectively silence disease-related genes through precise sequence matching 50-53. Since the discovery of RNAi in 1998 54, siRNA-based therapeutics have faced significant obstacles, including stability, delivery challenges, and potential off-target effects. However, continued advances in the field have culminated in the approval of pioneering RNAi therapies such as ONPATTRO® (Patisiran) 55 in 2018 and GIVLAARI™ (givosiran) 56, marking key milestones in the clinical application of siRNA. Unlike conventional therapeutic strategies using small molecules or monoclonal antibodies, which often require targeting protein structures or specific molecules, siRNA directly binds to mRNA via Watson-Crick base pairing, enabling gene silencing with high specificity 57, 58. This mechanism allows siRNA to avoid the need for detailed structural knowledge of protein targets, presenting a multifunctional therapeutic platform for a wide range of diseases, even those that are difficult to treat with traditional approaches. Theoretically, siRNA can be designed to target any gene, broadening its potential therapeutic applications and shortening the development timeline compared to other drugs.

### The challenges faced by therapeutic siRNA

Despite its promise, siRNA faces challenges, primarily related to its stability in the bloodstream, susceptibility to enzymatic degradation 59, and potential off-target gene silencing 60. Addressing these challenges has driven substantial progress in the chemical modification of siRNA molecules and the development of advanced delivery platforms. Modifications such as 2'-O-methylation 61 and locked nucleic acids (LNA) 62 have enhanced the stability, activity, and specificity of siRNA while reducing immune responses and off-target effects. Simultaneously, novel delivery systems-ranging from LNPs 63 and polymers 64 to peptides and exosomes have been explored to ensure targeted delivery of siRNA to the appropriate tissues, improving therapeutic outcomes. Recent breakthroughs have resulted in the clinical translation of siRNA-based therapies across a variety of medical fields. In metabolic disorders, nedosiran, a type of therapeutic siRNA, targets hepatic enzymes to treat primary hyperoxaluria, while cemdisiran, another siRNA drug, is being developed to inhibit complement 5 (C5) in rare complement-mediated diseases 65. Fitusiran is another siRNA drug aimed at reducing antithrombin to restore coagulation balance in hemophilia patients 66. Infectious disease applications include RG6346 67, which targets hepatitis B virus (HBV), and MIR 19, a potential treatment for Coronavirus Disease (COVID)-19. Oncology-focused siRNA therapies are targeting oncogenes in cancers like glioblastoma and mutant KRAS in pancreatic cancer 68. Ocular diseases such as age-related macular degeneration, diabetic macular edema, and glaucoma are being addressed with siRNA targeting vascular endothelial growth factor (VEGF) and its receptor. Moreover, siRNA is being developed to prevent acute kidney injury by inhibiting p53 69. The breadth of these applications underscores the emerging role of siRNA in modern medicine, with ongoing research likely to further expand its therapeutic potential.

### Utilizing PDNVs for therapeutic siRNA in disease therapy

PDNVs present several advantages for therapeutic siRNA delivery. They can efficiently encapsulate and protect siRNA from degradation, ensuring stability in biological environments. PDNVs have the inherent ability to cross biological barriers and can be targeted to specific tissues, making them ideal for precision medicine 70. Furthermore, their scalable production from plants allows for cost-effective and sustainable manufacturing, providing a green alternative to synthetic nanoparticles. These features highlight PDNVs as promising vectors in siRNA-based disease therapies.

Plant-derived exosome-like nanoparticles provide a compelling alternative, particularly for oral and nasal delivery. Li *et al.* demonstrated that folic acid-functionalized ginger-derived nanovesicles (GDENs) effectively delivered survivin siRNA to cancer cells, achieving significant gene knockdown *in vitro* and inhibiting tumor growth *in vivo*, highlighting the therapeutic potential and cost-effectiveness of PDNVs in cancer treatment 71. Sung *et al.* developed ginger-derived lipid nanoparticles loaded with CD98-siRNA, achieving targeted colon delivery and significant anti-inflammatory effects in a DSS-induced colitis mouse model, demonstrating the immunocompatibility and therapeutic efficacy of plant-derived systems for inflammatory disease treatment 72. Similarly, Cui *et al.* developed a ginger extracellular vesicle (GEV) and ZIF-8 nanoparticle system for TNF-α siRNA delivery, effectively targeting colon inflammation and reducing TNF-α levels *in vivo*. This approach highlights the potential of PDNVs for targeted siRNA delivery in ulcerative colitis treatment 73. Ganji *et al.* utilized tangerine-derived nanovesicles (TNVs) as carriers for DDHD1-siRNA, achieving 13% loading efficiency and 60% gene knockdown in colorectal cancer cells, demonstrating the feasibility of PDNVs for RNA interference therapies in mammalian disease treatment 74. Huang *et al.* used kiwi-derived extracellular vesicles (KEVs) as a cation-free platform to deliver STAT3 siRNA for EGFR-mutant NSCLC treatment. Modified with EGFR-targeting aptamers, these STAT3/EKEVs showed high stability, specificity, and effectiveness, significantly suppressing tumor growth in a mouse model through STAT3 inhibition 75. Itakura* et al.* developed a microfluidic device (MD) approach to encapsulate siRNA into grapefruit-derived vesicles (GEVs) with 11% encapsulation efficiency. The siRNA-loaded GEVs demonstrated effective delivery and gene suppression in human keratinocytes, highlighting GEVs as a safe, scalable alternative for siRNA delivery in gene therapy 76.

**Figure 3:**
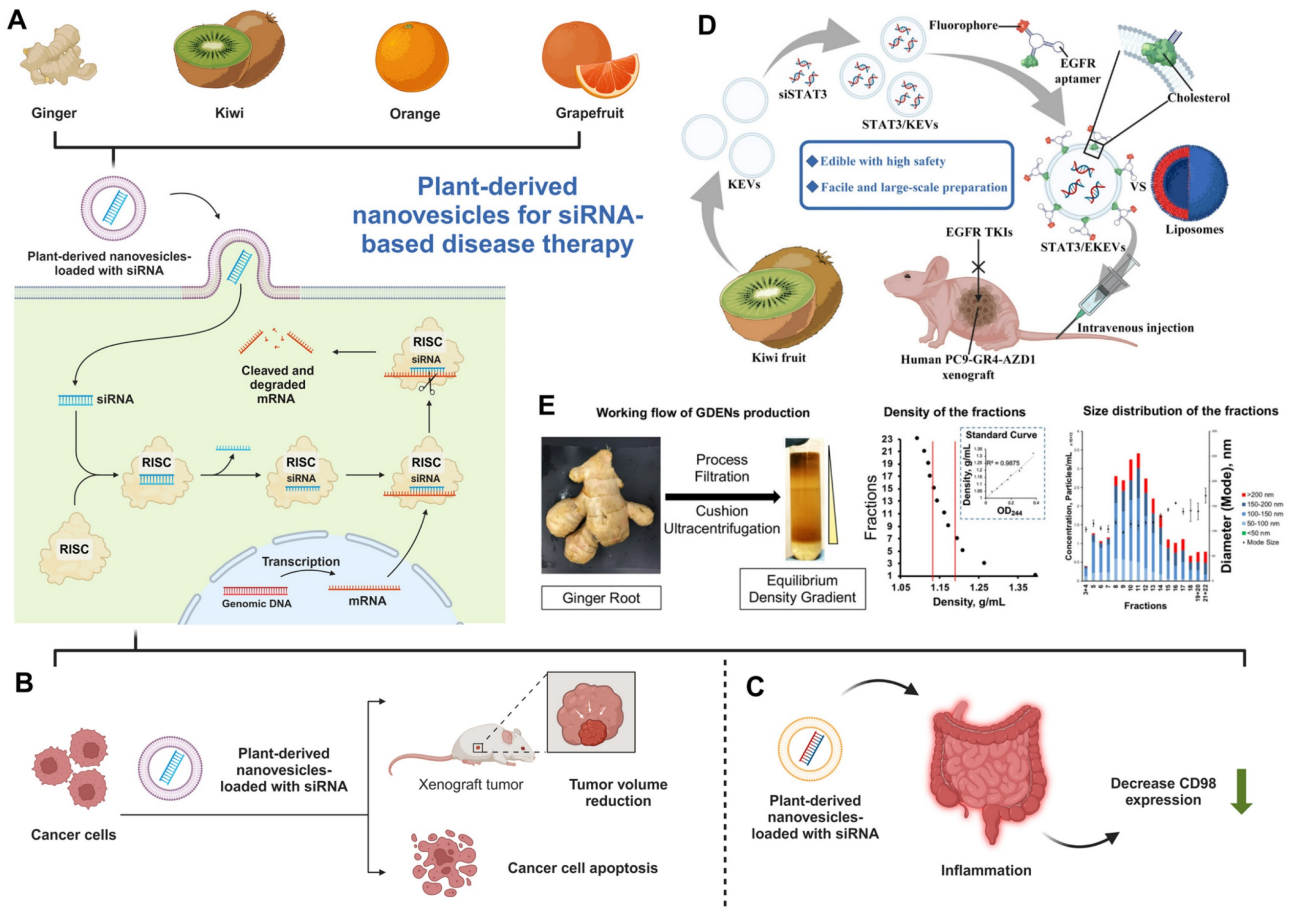
** PDNVs for siRNA-based disease therapy.** (A) Ginger, kiwi, orange, and grapefruit-derived nanovesicles for siRNA delivery system and the relevant mechanism for siRNA functions (created with biorender.com). (B-C) PDNVs are used for therapeutic siRNA in the therapy of (B) cancer and (C) inflammatory diseases (created with biorender.com). (D). A schematic representation of siSTAT3-loaded EKEVs (STAT3/EKEVs) designed for the effective treatment of PC9-GR4-AZD1 NSCLC tumor xenograft. (Reproduced with permission from Ref. 75: *Edible and cation-free kiwi fruit-derived vesicles mediated EGFR-targeted siRNA delivery to inhibit multidrug-resistant lung cancer*). Adapted from Huang *et al.*, Journal of Nanobiotechnology, 2023, under a Creative Commons CC BY license. (E). The purification and characterization of GDENs. (Reproduced with permission from Ref. 71: *Arrowtail RNA for Ligand Display on Ginger Exosome-like Nanovesicles to Systemic Deliver siRNA for Cancer Suppression*). Adapted from Li *et al.*, Scientific Reports, 2018, under a Creative Commons CC BY license.

PDNVs present a transformative solution for siRNA-based therapies (**Figure 3**), addressing key challenges in drug delivery such as biocompatibility, immunogenicity, and scalable production. Studies have demonstrated their capacity to efficiently deliver therapeutic siRNAs to targeted tissues, as seen with ginger-derived vesicles in cancer and inflammatory disease models. PDNVs offer a cost-effective, sustainable alternative to synthetic nanoparticles, with additional advantages such as natural bioactive compounds enhancing therapeutic efficacy. These properties position PDNVs as a promising next-generation platform for gene therapy applications.

## Leveraging PDNVs for miRNA therapeutics in disease treatment

### Biosynthesis and therapeutic potential of miRNAs

The identification of miRNAs by Victor Ambros's lab in 1993 77 revolutionized our understanding of gene regulation. miRNAs are small, non-coding RNA sequences (19-24 nucleotides) that regulate gene expression by binding to target mRNAs, resulting in post-transcriptional gene silencing 78, 79. This process is essential for controlling various cellular functions. miRNAs are generated from longer transcripts known as primary miRNAs (pri-miRNAs), which form hairpin structures from non-coding regions or introns. Most miRNAs are produced via a canonical pathway involving two key RNase III enzymes. The nuclear Microprocessor complex, comprising Drosha and DGCR8, processes pri-miRNA into precursor miRNA (pre-miRNA), a 55-70 nucleotide molecule. Pre-miRNAs are then transported to the cytoplasm, where Dicer cleaves them into a miRNA duplex. One strand of this duplex is incorporated into the RNA-induced silencing complex (RISC) by Argonaute (Ago) proteins, guiding gene repression 80-82. Recent advancements in miRNA research have paved the way for their therapeutic application, particularly in areas such as cancer 83, 84, cardiovascular diseases 85, and neurodegenerative disorders 86, 87. Therapeutic miRNAs can be designed to either inhibit overexpressed oncogenes or restore suppressed tumor-suppressor genes, demonstrating their dual potential in targeted gene regulation.

### Biomedical applications and challenges of miRNA therapeutics

miRNAs are pivotal in regulating gene expression and are implicated in numerous diseases, including immune disorders, Alzheimer's disease, cardiovascular conditions, rheumatoid arthritis, and various cancers. For instance, certain miRNAs regulate proteins involved in insulin signaling, linking them to insulin resistance and pancreatic cancer. Given their broad regulatory roles, miRNAs have emerged as promising therapeutic targets. Miravirsen 88, the first miRNA-based therapy targeting the hepatitis C virus (HCV), is undergoing phase II clinical trials, exemplifying its therapeutic potential.

Despite these advances, miRNA therapeutics face significant challenges. The primary issue is miRNA instability in biological systems due to nuclease-mediated degradation. Efficient, targeted delivery systems are also critical for the success of miRNA therapies. Delivery methods must ensure that miRNAs reach specific tissues or cells without inducing immune reactions. Moreover, off-target effects, where miRNAs inadvertently interact with unintended mRNAs, pose risks, potentially leading to adverse effects 89. Finally, extensive clinical trials and regulatory approvals are necessary to ensure the safety and efficacy of miRNA therapies, slowing their translation into clinical use.

### Harnessing PDNVs for miRNA-based therapeutics

PDNVs are emerging as highly promising tools for miRNA-based therapies. These natural vesicles not only function as carriers of exogenous miRNAs but also inherently contain bioactive miRNAs that can exert therapeutic effects. This dual functionality makes PDNVs a unique and effective platform for disease treatment. Their inherent miRNA content, derived from the host plant, can directly modulate biological pathways, while exogenously loaded miRNAs enhance their therapeutic versatility. The combination of biocompatibility, natural targeting capabilities, and protective properties positions PDNVs as next-generation vectors for delivering both intrinsic and therapeutic miRNAs.

#### Gastrointestinal tract applications

Broccoli-derived extracellular vesicles (BEVs) have been evaluated for delivering extracellular RNA. Pozo-Acebo *et al.* isolated BEVs using a combination of ultracentrifugation and size-exclusion chromatography, then loaded them with exogenous miRNAs. These miRNA-loaded BEVs were successfully taken up by Caco-2 cells, resulting in significant cytotoxicity at concentrations of 5% and above, with cell survival rates around 30%. This finding highlights BEVs' potential as effective delivery vehicles for exogenous miRNAs, offering advantages such as low immunogenicity and stability in the gastrointestinal environment, thus representing a promising approach for therapeutic applications 90. Nanovesicles like BEVs have shown resilience to harsh gastrointestinal conditions, including acidic pH and digestive enzymes, which often degrade other therapeutic agents. This stability makes PDNVs a promising approach for delivering miRNAs that require protection against degradation in the gastrointestinal tract, potentially enhancing bioavailability and efficacy. Garlic-derived exosome-like nanovesicles (GENs), containing 26 lipids, 61 proteins, and 127 miRNAs, were found to modulate inflammatory pathways. Specifically, han-miR3630-5p inhibited the toll-like receptor 4 (TLR4) pathway, reducing inflammatory cytokines in lipopolysaccharide (LPS)-induced Caco-2 cells and improving tight junction protein function. In colitis mouse models, GENs reduced histological damage and TLR4 signaling, highlighting their therapeutic potential for inflammatory bowel disease (IBD) 91.

#### Crossing the blood-brain barrier (BBB) for central nervous system therapies

Overcoming the BBB remains a significant challenge for central nervous system therapies due to the BBB's selective permeability. PDNVs offer unique advantages for crossing this barrier, as they can be functionalized and engineered to enhance targeted delivery while maintaining low immunogenicity. Grapefruit-derived nanovectors (GNVs) were explored by Zhuang *et al.* to deliver miR17 for treating brain tumors. By coating GNVs with folic acid (FA), they targeted folate receptor-positive GL-26 brain tumors. Combining FA-GNVs with polyethylenimine (FA-pGNVs) increased RNA-carrying capacity while reducing polyethylenimine toxicity. Intranasal delivery of miR17 using FA-pGNVs allowed rapid, targeted uptake in brain tumor cells, delaying tumor growth in mouse models, and indicating a promising noninvasive therapeutic method for brain diseases 92. Kim *et al.* also demonstrated the potential of ginseng-derived exosome-like nanoparticles for crossing the BBB, showing that these vesicles effectively delivered ptc-miR396f to C6 glioma cells. This miRNA silenced the c-MYC gene, suppressing tumor growth and enhancing survival in glioma-bearing mice. Additionally, ginseng-derived exosome-like nanoparticles modulated immune responses by reducing pro-tumoral cytokines, inducing T-cell activity, and suppressing regulatory T cells (Tregs), demonstrating their capacity to influence the tumor microenvironment (TME) 93. Together, these examples illustrate the ability of PDNVs to not only cross the BBB but also serve as highly specific, low-immunogenicity carriers for therapeutic agents in central nervous system diseases.

#### Cancer therapy applications

Corvigno *et al.* developed HEXPO, a delivery system using watermelon-derived PDNVs complexed with generation 3 PAMAM dendrimers to enhance small RNA delivery, such as miR146. The combination improved nucleic acid loading, increased *in vivo* safety, and prevented cellular membrane disruption. The anti-tumor efficacy of miR146 was demonstrated through its role in reducing angiogenesis and enhancing immune cell activation, particularly increasing CD8+ T-cell infiltration. This approach suggests the potential for combining plant-derived vesicle therapies with immune-targeted strategies for cancer treatment 94. Brucea javanica-derived nanovesicles (BF-Exos) were developed to target triple-negative breast cancer (TNBC), an aggressive cancer subtype. Yan *et al.* showed that BF-Exos delivered 10 functional miRNAs to 4T1 breast cancer cells, reducing cell proliferation and metastasis through regulation of the PI3K/Akt/mTOR pathway and ROS/caspase-mediated apoptosis. Additionally, BF-Exos reduced vascular endothelial growth factor secretion, limiting angiogenesis. In mouse models, BF-Exos inhibited tumor growth and metastasis, showing high biosafety and potential as a plant-derived therapeutic platform for TNBC 95.

#### Applications in inflammatory diseases

Similarly, ginger-derived exosome-like nanoparticles (GELNs) were explored by Yan *et al.*, where osa-miR164d was identified as a regulator of macrophage polarization. GELNs-loaded miR164d targeted TAB1, promoting M2 macrophage polarization, and reducing inflammation in intestinal tissues. Biomimetic exosomes loaded with osa-miR164d offer a scalable therapeutic option for inflammatory diseases 96.

**Figure 4:**
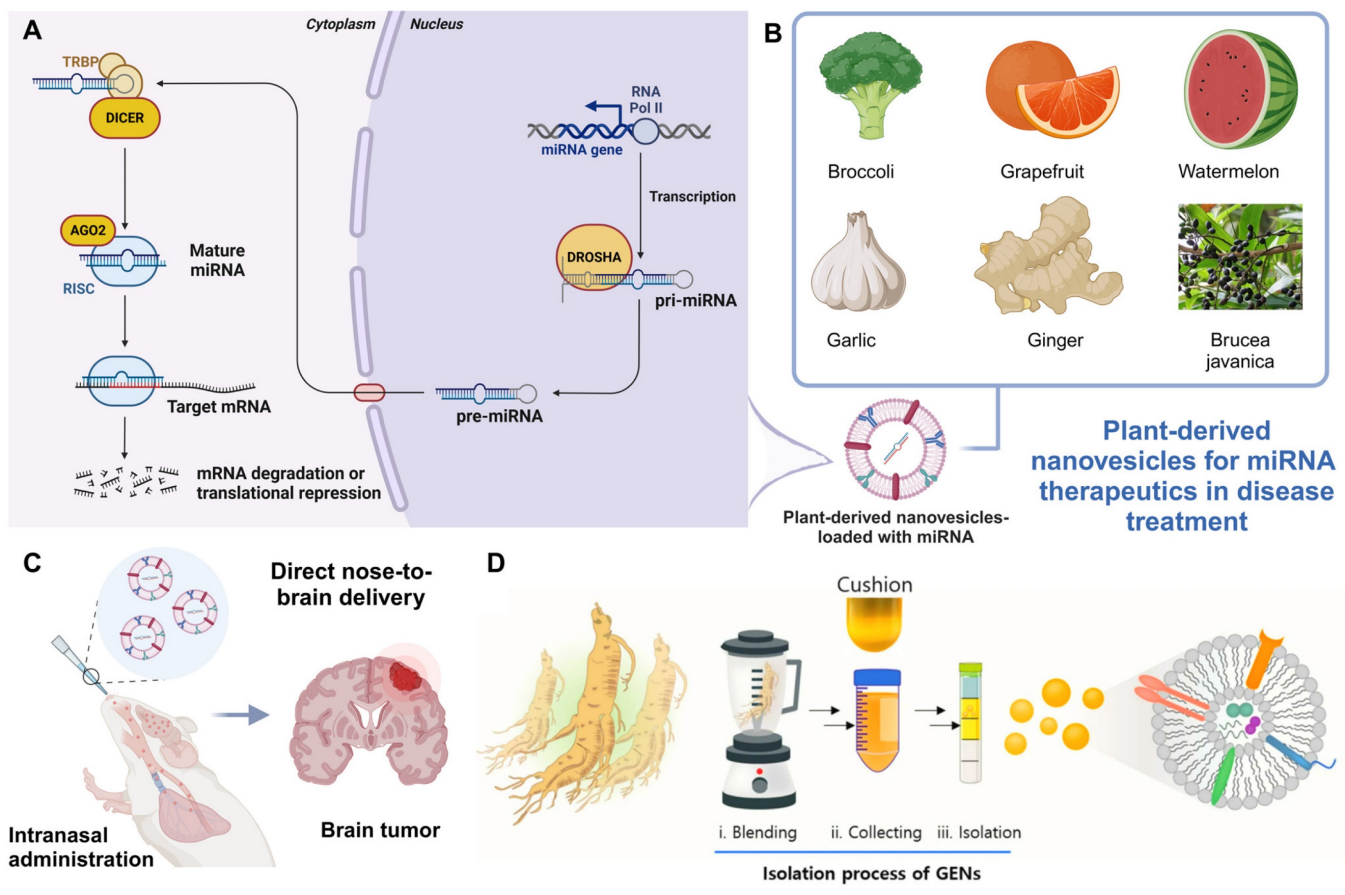
** Leveraging PDNVs for miRNA therapeutics in disease treatment**. (A) miRNA biogenesis and mechanism of action. The miRNA gene is transcribed by RNA polymerase II into pri-miRNA, which is processed by the Drosha complex into precursor miRNA (pre-miRNA). After being exported to the cytoplasm, Dicer further processes pre-miRNA into mature miRNA. This mature miRNA is incorporated into the RNA-induced silencing complex (RISC), where it guides AGO2 to the target mRNA, leading to mRNA degradation or translational repression (created with biorender.com). (B) Plant sources of nanovesicles for miRNA therapeutics (created with biorender.com). (C) PDNVs for delivering miRNA to the brain. Illustration of intranasal administration 92 of PDNVs for delivering miRNA directly to the brain, targeting brain tumors (created with biorender.com). (D) Isolation process of ginseng-derived nanovesicles. (Reproduced with permission from Ref. 93: *Anti-glioma effect of ginseng-derived exosomes-like nanoparticles by active blood-brain-barrier penetration and tumor microenvironment modulation*). Adapted from Kim *et al.*, Journal of Nanobiotechnology, 2023, under a Creative Commons CC BY license.

This section discusses recent advancements in PDNVs as miRNA delivery systems for therapeutic applications (**Figure 4**). PDNVs, such as those derived from broccoli, grapefruit, watermelon, garlic, ginger, and Brucea javanica, provide enhanced stability, targeting abilities, and biocompatibility. These nanovesicles have been shown to effectively deliver miRNAs across various barriers, including the gastrointestinal tract and BBB, making them promising vehicles for treating conditions such as cancer, inflammation, and brain tumors. Studies highlight the role of miRNAs in regulating key signaling pathways, such as PI3K/Akt/mTOR, TLR4, and NF-κB, leading to tumor suppression, immune activation, and inflammation reduction. The combination of PDNVs with other delivery technologies, such as PAMAM dendrimers and biomimetic exosomes, further enhances therapeutic efficacy, offering innovative solutions for gene therapy in cancer and inflammatory diseases.

## PDNVs as emerging platforms for other gene therapies

Gene therapy is revolutionizing modern medicine, offering innovative approaches to treat a broad spectrum of diseases. Despite these advancements, the efficient and targeted delivery of therapeutic nucleic acids remains a significant obstacle. PDNVs have emerged as promising biocompatible carriers for gene therapies, including the delivery of mRNA, DNA, and other therapeutic agents (**Table 1**). PDNVs offer distinct advantages such as enhanced nucleic acid stability, reduced immunogenicity, and potential for tissue-specific targeting. This section discusses how PDNVs are addressing limitations in nucleic acid-based therapies and their potential to reshape the landscape of gene therapy.

### PDNVs for mRNA therapeutics

mRNA therapeutics have rapidly gained prominence, particularly after the success of mRNA-based COVID-19 vaccines 97-100. However, the widespread use of mRNA therapies is hindered by challenges such as mRNA instability, enzymatic degradation, and immune reactions triggered by current delivery systems like LNPs 101, 102. PDNVs offer a natural, biocompatible solution to these issues. Unlike conventional mRNA carriers, PDNVs provide robust protection to encapsulated mRNA, enhancing both its stability and bioavailability. Their low immunogenicity mitigates the risk of unwanted immune responses, while their ability to be engineered for tissue-specific targeting improves delivery efficiency. Furthermore, the scalability and cost-effectiveness of PDNV production make them a compelling alternative for mRNA-based treatments. For instance, Pomatto *et al.* demonstrated the potential of PDNVs derived from Citrus sinensis juice to deliver an oral mRNA vaccine targeting the SARS-CoV-2 S1 protein. The PDNV-encapsulated mRNA vaccine remained stable at room temperature for up to a year and successfully triggered both systemic and mucosal immune responses in rats following oral administration. This study highlights the potential of PDNVs to not only improve the stability of mRNA but also pave the way for non-invasive vaccine delivery methods, such as oral administration, which overcome the logistical challenges of cold-chain storage associated with traditional vaccines 103.

### PDNVs for DNA-based gene therapy

DNA-based gene therapy has advanced significantly, particularly with technologies like recombinant DNA 104, viral vectors 105, and CRISPR-Cas9 106. Despite these achievements, delivery efficiency, potential cytotoxicity, and immune reactions to foreign DNA remain persistent hurdles 107. PDNVs have emerged as effective carriers for therapeutic DNA, addressing many of these limitations. PDNVs enhance the stability and delivery efficiency of therapeutic DNA while reducing immunogenicity and cytotoxicity. Their ability to encapsulate nucleic acids and be modified for targeted delivery ensures precise gene therapy with minimal off-target effects. For example, Wang *et al.* demonstrated the use of GNVs to deliver a biotinylated eYFP DNA expression vector into A549 cells. The study showed that protein expression was comparable to conventional methods like Lipofectamine 2000, while GNVs avoided the cytotoxicity typically seen with synthetic carriers. Furthermore, the study demonstrated successful encapsulation and delivery of the luciferase gene in CD4+ and CD8+ T cells without triggering inflammatory responses. These findings position PDNVs as a versatile and safer alternative to traditional delivery systems, with potential applications across various DNA-based therapies 108.

### PDNVs in other emerging gene therapies

As gene therapy continues to evolve, novel nucleic acid-based strategies are emerging, offering new therapeutic possibilities. PDNVs have demonstrated their versatility not only in delivering well-established therapeutic agents such as mRNA, DNA, siRNA, and miRNA, but also in their potential to serve as carriers for other types of nucleic acids, including antisense oligonucleotides (ASOs), aptamers, and circular RNA (circRNA). These newer therapeutic approaches, when combined with the biocompatibility and efficiency of PDNVs, offer exciting prospects for future gene therapies.

As the field of gene therapy continues to evolve, new technologies such as CRISPR-Cas9 and PDNVs are pushing the boundaries of treatment possibilities 109. CRISPR-Cas9 enables precise genome editing, allowing targeted correction of genetic mutations or inactivation of harmful genes. Coupling CRISPR with PDNVs as delivery vehicles offers a promising approach to enhance the safety and efficacy of genome editing, leveraging the biocompatibility, targeting capacity, and low immunogenicity of PDNVs. This combination is particularly valuable for treating genetic disorders, such as cystic fibrosis, muscular dystrophy, and cancer, by improving delivery precision and reducing off-target effects. Together, CRISPR and PDNVs represent a significant step forward in the development of next-generation gene therapies.

ASOs are short, single-stranded DNA or RNA molecules designed to bind to specific mRNA transcripts, preventing the translation of target genes 110. This approach holds promise for treating genetic disorders caused by aberrant or harmful gene expression, such as spinal muscular atrophy (SMA) 111 or Duchenne muscular dystrophy (DMD) 112. However, ASO therapies face similar challenges to other nucleic acid-based treatments, such as instability and off-target effects. PDNVs could serve as highly effective carriers for ASOs, enhancing their stability in biological environments and improving delivery efficiency to specific tissues, thus maximizing therapeutic outcomes while minimizing side effects.

Aptamers are short, single-stranded oligonucleotides that fold into specific three-dimensional shapes, allowing them to bind tightly and specifically to target molecules, much like antibodies 113. These nucleic acid-based ligands have potential applications in diagnostics, drug delivery, and targeted therapies, particularly for cancer and infectious diseases. When combined with PDNVs, aptamers could benefit from enhanced stability and protection from degradation, while the natural targeting capabilities of PDNVs could be exploited to further improve the specificity and efficiency of aptamer-based treatments.

CircRNA is a type of non-coding RNA that forms a covalently closed loop, making it more stable than linear RNA species 114. Due to its resistance to exonucleases, circRNA has gained interest as a potential therapeutic agent, particularly for gene regulation and protein production. PDNVs could provide an optimal delivery system for circRNAs, enhancing their stability and enabling tissue-specific targeting. Given the recent interest in circRNA for applications such as cancer therapy and regenerative medicine, PDNVs could play a crucial role in advancing circRNA-based treatments by overcoming the challenges associated with delivery and stability.

In addition to the established applications, researchers are increasingly exploring combination therapies involving multiple nucleic acids (e.g., combining miRNA and siRNA or using mRNA and ASOs together). PDNVs offer a flexible platform for such combination strategies, potentially enabling the co-delivery of different nucleic acids to achieve synergistic effects in gene regulation or disease treatment. For example, co-delivering miRNA and siRNA could simultaneously target multiple pathways in cancer therapy 115, 116.

## Challenges and perspectives

### Current limitations and potential solutions

#### Isolation and purification

PDNVs present several unique challenges that need to be addressed to fully realize their potential as gene-delivery vehicles. One significant challenge lies in the isolation and standardization of PDNVs. The extraction and purification processes vary significantly depending on the plant source and the methodology used, which can lead to inconsistencies in yield, purity, and biological activity 118, 119. This lack of standardization poses a barrier to large-scale production and clinical translation. Additionally, the characterization and quality control of PDNVs are complex 120. Ensuring consistent properties such as size, surface charge, and cargo content is crucial but technically challenging. Advanced analytical techniques are required to fully characterize PDNVs and meet regulatory standards. Additionally, the development of more efficient extraction and purification techniques, such as affinity-based purification methods, could enhance PDNVs yield and purity. Standardizing extraction protocols across labs to improve batch consistency would also support reproducibility in experimental and therapeutic applications (**Table 2**).

#### Loading and stabilization

Another major challenge is the efficient loading and stabilization of therapeutic nucleic acids within PDNVs. Encapsulating exogenous nucleic acids like siRNA, miRNA, mRNA, or DNA without compromising the structural integrity of the vesicles is difficult. Furthermore, maintaining the stability of these nucleic acids during storage, transport, and after administration is essential for therapeutic efficacy. Therefore, optimizing loading techniques, perhaps by exploring lipid modification strategies or co-encapsulation with stabilizing agents, could improve encapsulation efficiency and nucleic acid stability within PDNVs.

Efficiently loading nucleic acids into PDNVs remains a significant challenge compared to other delivery systems like LNPs and EVs. Traditional methods, including electroporation, sonication, and hybridization approaches, each have unique advantages and limitations for PDNVs. For instance, electroporation can facilitate nucleic acid loading by transiently permeabilizing PDNV membranes; however, it may compromise vesicle integrity if not carefully optimized. Sonication, another common approach, can enhance encapsulation efficiency but may disrupt PDNV structure and lead to inconsistent loading. Hybridization approaches, where nucleic acids are conjugated or hybridized to vesicle surfaces, offer a gentler alternative but may result in lower encapsulation efficiency and stability. Recent studies have begun to explore modified methods, such as combining electroporation with stabilizing agents 71, 103 or developing hybrid techniques 92 that maintain PDNV integrity while enhancing loading capacity. These strategies underscore the need for tailored approaches to overcome the unique loading challenges in PDNVs, paving the way for more efficient nucleic acid delivery in therapeutic applications.

#### Targeted delivery, tissue specificity, and administration methods

Efficient delivery and cellular uptake of nucleic acids are critical for their therapeutic action. PDNVs must be engineered to improve cellular uptake and facilitate endosomal escape, ensuring the release of the therapeutic cargo into the cytoplasm. Off-target effects pose another major challenge, as unintended gene silencing or activation can lead to adverse effects. Furthermore, achieving precise targeting and biodistribution of PDNVs is also a significant hurdle. Although PDNVs can cross biological barriers, their ability to deliver cargo specifically to target tissues or cells needs improvement. Without appropriate targeting, off-target effects could reduce therapeutic efficacy and potentially cause side effects. Therefore, engineering surface modifications (e.g., with targeting ligands or peptides) on PDNVs could enhance tissue specificity. Additionally, leveraging plant-derived characteristics for targeting could be an area of innovative exploration.

The administration route of PDNVs further influences drug delivery efficiency and therapeutic outcomes. For example, oral administration is favorable for patient compliance but requires PDNVs to resist gastrointestinal enzymes and pH changes. Potential solutions include coating PDNVs with gastro-protective polymers or encapsulating them within resistant lipid formulations to maintain drug integrity. Intravenous administration allows for rapid systemic distribution but requires PDNVs to evade immune detection. Strategies to achieve this include PEGylation or cloaking PDNVs with biomimetic cell membranes to improve circulation time and reduce immune clearance. Intranasal delivery is a non-invasive option that provides access to the central nervous system, bypassing the blood-brain barrier. Enhancing the efficiency may involve engineering PDNVs with mucosal adhesive molecules or optimizing vesicle size for better nasal absorption. Together, selecting the appropriate administration method and incorporating targeted delivery strategies could significantly enhance PDNV delivery, addressing key challenges and maximizing therapeutic outcomes.

#### Immune response and biocompatibility

Immune response and biocompatibility are additional concerns. Although PDNVs are generally regarded as biocompatible and less immunogenic compared to synthetic nanoparticles, variations in their composition might elicit immune responses. Comprehensive preclinical studies are necessary to fully evaluate their safety profile, particularly for long-term applications. On the nucleic acid side, stability and degradation remain significant issues. RNA-based therapeutics are particularly susceptible to enzymatic degradation, which can limit their therapeutic potential 121, 122. Developing PDNV formulations that protect these nucleic acids from degradation and enhance their stability is essential. Besides, systematic studies to evaluate the immunogenic profile of PDNVs are necessary. Modifying PDNV membranes or pre-conditioning them to reduce immune recognition could enhance biocompatibility.

#### Regulatory and standardization issues

As a relatively new technology, PDNVs face significant regulatory challenges, with limited standards for their therapeutic use in gene therapy, making clinical translation more challenging. And regulatory hurdles add another layer of complexity. The approval process for gene therapies is strict, and addressing the standardization, safety, and efficacy of PDNV-based therapies will require robust preclinical data and well-designed clinical trials. Therefore, establishing regulatory guidelines and quality standards specific to PDNVs could facilitate their path to clinical applications. Collaboration between research and regulatory bodies may help in developing frameworks for safe and effective PDNV-based therapies.

Additionally, a significant challenge in the large-scale production and clinical application of PDNVs is batch-to-batch variability, which can arise from factors such as plant species, seasonal variations, and cultivation conditions. These variables can impact the size, composition, and yield of PDNVs, introducing inconsistencies that hinder reproducibility and scalability. Currently, due to the small-scale production of most PDNV research, few methods are available to address these issues directly. However, exploring standardized cultivation practices and advanced purification protocols shows promise for reducing variability. For example, controlled growth environments and genetically engineered plant lines are being investigated to produce more uniform PDNVs with consistent therapeutic profiles. Quality control measures, such as regular biochemical profiling and the use of molecular markers, are also being developed to monitor and ensure the consistency of PDNV batches. These approaches are paving the way for more reliable and scalable production, essential for clinical applications.

### Perspectives

Despite these challenges, there are several promising avenues for advancing PDNV-based gene therapy. Improving the methods for isolating and purifying PDNVs is a critical first step. Developing more efficient, scalable, and standardized processes will enhance the reproducibility and quality of PDNVs, facilitating their clinical use. Additionally, surface modification techniques can be employed to functionalize PDNVs by targeting ligands 123, peptides, or antibodies, thereby improving their specificity for diseased cells or tissues 70. Such modifications could significantly enhance the therapeutic efficacy of PDNVs and minimize off-target effects. Hybrid PDNV formulations, which combine natural and synthetic materials, offer another promising strategy. These hybrid systems could leverage the biocompatibility of PDNVs and the robustness of synthetic carriers to create more versatile delivery platforms. In terms of nucleic acid modifications, integrating chemical modifications like 2'-O-methylation or phosphorothioate linkages could improve the stability and reduce the immunogenicity of the therapeutic cargo. Co-delivery strategies that combine multiple types of nucleic acids, such as miRNA and mRNA, within a single PDNV formulation could provide synergistic therapeutic effects, addressing multiple disease pathways simultaneously.

Expanding the therapeutic applications of PDNV-based gene therapies is another exciting perspective. While much of the current research focuses on cancer and inflammatory diseases, there is potential for PDNVs in regenerative medicine, neurological disorders, and metabolic diseases. A deeper understanding of PDNV biodistribution and their interaction with various tissues will enable the development of more targeted and effective therapies for these conditions. On the regulatory and clinical front, establishing clear guidelines for PDNV-based therapies will be essential. Collaboration between researchers, industry stakeholders, and regulatory agencies can help develop standardized protocols for the production, characterization, and clinical evaluation of PDNVs. Comprehensive preclinical studies focusing on the pharmacokinetics, biodistribution, and safety of PDNVs are needed to support clinical trials. Early-phase clinical trials should prioritize the assessment of safety and preliminary efficacy in specific diseases to establish a foundation for broader applications. Engaging with the public and addressing ethical considerations are also important. Public perception and ethical concerns regarding the use of plant-derived materials must be managed to facilitate the acceptance of PDNV-based gene therapies. Discussing potential environmental impacts and demonstrating the safety and efficacy of these therapies will be crucial for gaining public trust.

In conclusion, PDNVs offer a promising and biocompatible platform for nucleic acid-based gene therapy, with the potential to revolutionize the treatment of various medical conditions. Addressing the current challenges through advances in isolation techniques, surface modification, and hybrid formulations, along with robust regulatory and clinical efforts, will be key to unlocking their full therapeutic potential. Continued research and multidisciplinary collaboration will be essential to advance PDNV-based therapies into next-generation gene therapy solutions.

## Conclusion

In this review, we have comprehensively explored the potential of PDNVs as innovative delivery systems for nucleic acid-based gene therapies. As the field of gene therapy evolves beyond traditional methods of direct gene correction, the focus has shifted towards employing nucleic acids such as mRNA, miRNA, siRNA, and DNA to modulate gene expression and deliver functional genetic material without altering the genome. We have highlighted the unique advantages of PDNVs in addressing the limitations of conventional delivery systems, including their biocompatibility, low immunogenicity, and natural targeting capabilities.

We began by discussing the biological origins and properties of PDNVs, emphasizing their production from various plant sources like ginger, grapefruit, garlic, and ginseng. These vesicles have shown the ability to encapsulate and protect therapeutic nucleic acids, enhancing their stability and bioavailability in biological environments. The review also detailed the isolation and purification processes for PDNVs, along with their inherent challenges in standardization and quality control. In the context of gene therapy, we examined the current applications of PDNVs in delivering different nucleic acids for the treatment of a wide range of diseases, including cancer, inflammatory conditions, and genetic disorders. Studies on siRNA delivery using PDNVs have demonstrated promising results in cancer therapy and inflammatory disease models. Similarly, PDNVs have been investigated as carriers for miRNA therapeutics, showing potential in regulating key signaling pathways involved in disease progression. We also explored the emerging applications of PDNVs for delivering mRNA and DNA, discussing their potential in areas such as vaccine development and genetic disease treatment.

Despite the promising data, several challenges remain. The review identified key issues such as the variability in PDNV isolation methods, the need for efficient and stable nucleic acid loading, and achieving specific targeting and biodistribution. We also addressed the immunogenicity and safety concerns associated with PDNVs, which are crucial for their clinical translation. Looking forward, we discussed perspectives for advancing PDNV-based gene therapy. Enhancements in isolation and purification techniques, the development of hybrid PDNV formulations, and surface modifications for targeted delivery are promising strategies to overcome current limitations. Additionally, innovations in nucleic acid design and the co-delivery of multiple therapeutic agents could further expand the therapeutic scope of PDNVs. Regulatory and clinical efforts will be essential to establish standardized protocols and ensure the safe and effective use of these novel delivery systems.

In conclusion, this review underscores the transformative potential of PDNVs in nucleic acid-based gene therapies. By leveraging the natural properties of plant-derived vesicles, PDNVs offer a promising platform that combines biocompatibility with effective gene delivery. As research progresses, multidisciplinary collaboration and technological advancements will be crucial to unlock the full potential of PDNVs, paving the way for next-generation gene therapy solutions that are safer, more effective, and accessible for a broader range of diseases.

## Figures and Tables

**Table 1:** PDNVs for nucleic acid-based disease therapy.

Plant sources	Nucleic acid	Disease	Administration route	Reference
Ginger	siRNA	Cancer	Intravenous	71
	siRNA	Dextran Sodium Sulfate (DSS)-induced colitis	Oral	72
	siRNA	Ulcerative colitis	Oral	73
	miRNA	Intestinal inflammation	Oral	96
Orange	siRNA	Colorectal cancer	/	74
	mRNA	Infectious disease	Oral	103
	mRNA	Infectious disease	Oral or Intranasal	117
Kiwi	siRNA	Non-small cell lung cancer	Intravenous	75
Grapefruit	miRNA	Brain tumor	Intranasal	92
	DNA	Cancer	Intravenous	108
Watermelon	miRNA	Cancer	Intraperitoneal	94
Garlic	miRNA	Inflammatory bowel disease	Oral	91
Ginseng	miRNA	Glioma	Intravenous or Intracerebral	93
Brucea javanica	miRNA	Triple-negative breast cancer	Intratumoral	95

**Table 2:** Challenges of PDNVs extraction and purification methods.

Method	Guideline	Advantage	Challenge
Ultracentrifugation	Size and density	Simple separating Large sample processing Preserving integrity	Time-consuming Requiring specialized equipment Damaging particles
Dialysis	Removing small molecule impurities	Removing small impurities effectively	Relatively time-intensive Lower efficiency
Size-exclusion chromatography	Size	High purity of isolated PDNVs Shorter Processing time Preserves EV integrity and biological activity	High cost Limited sample volume
Immunoaffinity capture-based technique	Selective enrichment using specific covalent or affinity magnetic beads	Higher extraction purity Higher yield Suitable for large diluted samples	Longer processing time Longer processing time Higher extraction cost
Differential ultrafiltration	Size	Allowing handling of larger sample volumes	Limited by particle size Affected recovery rate

## References

[1] Bulaklak K, Gersbach CA (2020). The once and future gene therapy. Nat Commun.

[2] Liu J, Lu X, Wu T, Wu X, Han L, Ding B (2021). Branched Antisense and siRNA Co-Assembled Nanoplatform for Combined Gene Silencing and Tumor Therapy. Angew Chem Int Ed Engl.

[3] Zhao Y, Zhao T, Cao Y, Sun J, Zhou Q, Chen H (2021). Temperature-Sensitive Lipid-Coated Carbon Nanotubes for Synergistic Photothermal Therapy and Gene Therapy. ACS Nano.

[4] Zhu Y, Shen R, Vuong I, Reynolds RA, Shears MJ, Yao ZC (2022). Multi-step screening of DNA/lipid nanoparticles and co-delivery with siRNA to enhance and prolong gene expression. Nat Commun.

[5] Lin C, Teng C, Li B, He W Anti-inflammatory drug-assisted microRNA gene therapy for effectively improving pulmonary hemodynamics. Chinese Chemical Letters. 2024: 110450.

[6] Ho PTB, Clark IM, Le LTT (2022). MicroRNA-Based Diagnosis and Therapy. Int J Mol Sci.

[7] Ma Y, Lin H, Wang P, Yang H, Yu J, Tian H (2023). A miRNA-based gene therapy nanodrug synergistically enhances pro-inflammatory antitumor immunity against melanoma. Acta Biomater.

[8] Banoun H (2023). mRNA: Vaccine or Gene Therapy? The Safety Regulatory Issues. Int J Mol Sci.

[9] Gurumurthy CB, Quadros RM, Ohtsuka M (2022). Prototype mouse models for researching SEND-based mRNA delivery and gene therapy. Nat Protoc.

[10] Li Z, Zhao P, Zhang Y, Wang J, Wang C, Liu Y (2021). Exosome-based Ldlr gene therapy for familial hypercholesterolemia in a mouse model. Theranostics.

[11] He M, He M, Nie C, Yi J, Zhang J, Chen T (2021). mRNA-Activated Multifunctional DNAzyme Nanotweezer for Intracellular mRNA Sensing and Gene Therapy. ACS Appl Mater Interfaces.

[12] Gao Y, Men K, Pan C, Li J, Wu J, Chen X (2021). Functionalized DMP-039 Hybrid Nanoparticle as a Novel mRNA Vector for Efficient Cancer Suicide Gene Therapy. Int J Nanomedicine.

[13] Wang D, Yi H, Geng S, Jiang C, Liu J, Duan J (2023). Photoactivated DNA Nanodrugs Damage Mitochondria to Improve Gene Therapy for Reversing Chemoresistance. ACS Nano.

[14] Tang W, Tong T, Wang H, Lu X, Yang C, Wu Y (2023). A DNA Origami-Based Gene Editing System for Efficient Gene Therapy in Vivo. Angew Chem Int Ed Engl.

[15] Wu X, Liu Q, Liu F, Wu T, Shang Y, Liu J (2021). An RNA/DNA hybrid origami-based nanoplatform for efficient gene therapy. Nanoscale.

[16] Tang W, Han L, Duan S, Lu X, Wang Y, Wu X (2021). An Aptamer-Modified DNA Tetrahedron-Based Nanogel for Combined Chemo/Gene Therapy of Multidrug-Resistant Tumors. ACS Appl Bio Mater.

[17] Ke X, Shelton L, Hu Y, Zhu Y, Chow E, Tang H (2020). Surface-Functionalized PEGylated Nanoparticles Deliver Messenger RNA to Pulmonary Immune Cells. ACS Appl Mater Interfaces.

[18] Tschaharganeh D, Ehemann V, Nussbaum T, Schirmacher P, Breuhahn K (2007). Non-specific effects of siRNAs on tumor cells with implications on therapeutic applicability using RNA interference. Pathol Oncol Res.

[19] Yang Z, Liu W, Zhao L, Yin D, Feng J, Li L (2023). Single-exonuclease nanocircuits reveal the RNA degradation dynamics of PNPase and demonstrate potential for RNA sequencing. Nat Commun.

[20] Ding L, Li J, Wu C, Yan F, Li X, Zhang S (2020). A self-assembled RNA-triple helix hydrogel drug delivery system targeting triple-negative breast cancer. J Mater Chem B.

[21] Ghosh S, Brown AM, Jenkins C, Campbell K (2020). Viral Vector Systems for Gene Therapy: A Comprehensive Literature Review of Progress and Biosafety Challenges. Appl Biosaf.

[22] Brown AM, Blind J, Campbell K, Ghosh S (2020). Safeguards for Using Viral Vector Systems in Human Gene Therapy: A Resource for Biosafety Professionals Mitigating Risks in Health Care Settings. Appl Biosaf.

[23] Halman JR, Kim KT, Gwak SJ, Pace R, Johnson MB, Chandler MR (2020). A cationic amphiphilic co-polymer as a carrier of nucleic acid nanoparticles (Nanps) for controlled gene silencing, immunostimulation, and biodistribution. Nanomedicine.

[24] Miyazaki T, Uchida S, Nagatoishi S, Koji K, Hong T, Fukushima S (2020). Polymeric Nanocarriers with Controlled Chain Flexibility Boost mRNA Delivery In Vivo through Enhanced Structural Fastening. Adv Healthc Mater.

[25] Oh J, Kim SM, Lee EH, Kim M, Lee Y, Ko SH (2020). Messenger RNA/polymeric carrier nanoparticles for delivery of heme oxygenase-1 gene in the post-ischemic brain. Biomater Sci.

[26] Vysochinskaya V, Shishlyannikov S, Zabrodskaya Y, Shmendel E, Klotchenko S, Dobrovolskaya O (2022). Influence of Lipid Composition of Cationic Liposomes 2X3-DOPE on mRNA Delivery into Eukaryotic Cells. Pharmaceutics.

[27] Sanchez-Meza LV, Bello-Rios C, Eloy JO, Gomez-Gomez Y, Leyva-Vazquez MA, Petrilli R (2024). Cationic Liposomes Carrying HPV16 E6-siRNA Inhibit the Proliferation, Migration, and Invasion of Cervical Cancer Cells. Pharmaceutics.

[28] Cheng Q, Wei T, Farbiak L, Johnson LT, Dilliard SA, Siegwart DJ (2020). Selective organ targeting (SORT) nanoparticles for tissue-specific mRNA delivery and CRISPR-Cas gene editing. Nat Nanotechnol.

[29] Dilliard SA, Cheng Q, Siegwart DJ (2021). On the mechanism of tissue-specific mRNA delivery by selective organ targeting nanoparticles. Proc Natl Acad Sci U S A.

[30] Zimmermann CM, Baldassi D, Chan K, Adams NBP, Neumann A, Porras-Gonzalez DL (2022). Spray drying siRNA-lipid nanoparticles for dry powder pulmonary delivery. J Control Release.

[31] Ou X, Wang H, Tie H, Liao J, Luo Y, Huang W (2023). Novel plant-derived exosome-like nanovesicles from Catharanthus roseus: preparation, characterization, and immunostimulatory effect via TNF-alpha/NF-kappaB/PU.1 axis. J Nanobiotechnology.

[32] Wolf P (1967). The nature and significance of platelet products in human plasma. Br J Haematol.

[33] Zhuang X, Deng ZB, Mu J, Zhang L, Yan J, Miller D (2015). Ginger-derived nanoparticles protect against alcohol-induced liver damage. J Extracell Vesicles.

[34] Fang Z, Liu K (2022). Plant-derived extracellular vesicles as oral drug delivery carriers. J Control Release.

[35] Savci Y, Kirbas OK, Bozkurt BT, Abdik EA, Tasli PN, Sahin F (2021). Grapefruit-derived extracellular vesicles as a promising cell-free therapeutic tool for wound healing. Food Funct.

[36] Niu W, Xiao Q, Wang X, Zhu J, Li J, Liang X (2021). A Biomimetic Drug Delivery System by Integrating Grapefruit Extracellular Vesicles and Doxorubicin-Loaded Heparin-Based Nanoparticles for Glioma Therapy. Nano Lett.

[37] Long F, Pan Y, Li J, Sha S, Shi X, Guo H (2023). Orange-derived extracellular vesicles nanodrugs for efficient treatment of ovarian cancer assisted by transcytosis effect. Acta Pharm Sin B.

[38] Jang E, Yu H, Kim E, Hwang J, Yoo J, Choi J (2024). The Therapeutic Effects of Blueberry-Treated Stem Cell-Derived Extracellular Vesicles in Ischemic Stroke. Int J Mol Sci.

[39] Han X, Wei Q, Lv Y, Weng L, Huang H, Wei Q (2022). Ginseng-derived nanoparticles potentiate immune checkpoint antibody efficacy by reprogramming the cold tumor microenvironment. Mol Ther.

[40] Gao C, Zhou Y, Chen Z, Li H, Xiao Y, Hao W (2022). Turmeric-derived nanovesicles as novel nanobiologics for targeted therapy of ulcerative colitis. Theranostics.

[41] Tan X, Xu Y, Zhou S, Pan M, Cao Y, Cai X (2023). Advances in the Study of Plant-Derived Vesicle-Like Nanoparticles in Inflammatory Diseases. J Inflamm Res.

[42] Feng J, Xiu Q, Huang Y, Troyer Z, Li B, Zheng L (2023). Plant-Derived Vesicle-Like Nanoparticles as Promising Biotherapeutic Tools: Present and Future. Adv Mater.

[43] Wang X, Xin C, Zhou Y, Sun T (2024). Plant-Derived Vesicle-like Nanoparticles: The Next-Generation Drug Delivery Nanoplatforms. Pharmaceutics.

[44] Tinnirello V, Rabienezhad Ganji N, De Marcos Lousa C, Alessandro R, Raimondo S (2023). Exploiting the Opportunity to Use Plant-Derived Nanoparticles as Delivery Vehicles. Plants (Basel).

[45] Man F, Meng C, Liu Y, Wang Y, Zhou Y, Ma J (2021). The Study of Ginger-Derived Extracellular Vesicles as a Natural Nanoscale Drug Carrier and Their Intestinal Absorption in Rats. AAPS PharmSciTech.

[46] Wang Q, Ren Y, Mu J, Egilmez NK, Zhuang X, Deng Z (2015). Grapefruit-Derived Nanovectors Use an Activated Leukocyte Trafficking Pathway to Deliver Therapeutic Agents to Inflammatory Tumor Sites. Cancer Res.

[47] Wen HG, Zhao JH, Zhang BS, Gao F, Wu XM, Yan YS (2023). Microbe-induced gene silencing boosts crop protection against soil-borne fungal pathogens. Nat Plants.

[48] Wall NR, Shi Y (2003). Small RNA: can RNA interference be exploited for therapy?. Lancet.

[49] Mello CC, Conte D Jr (2004). Revealing the world of RNA interference. Nature.

[50] Huang X, Liu C, Kong N, Xiao Y, Yurdagul A Jr, Tabas I (2022). Synthesis of siRNA nanoparticles to silence plaque-destabilizing gene in atherosclerotic lesional macrophages. Nat Protoc.

[51] El Moukhtari SH, Garbayo E, Amundarain A, Pascual-Gil S, Carrasco-Leon A, Prosper F (2023). Lipid nanoparticles for siRNA delivery in cancer treatment. J Control Release.

[52] Li S, Li J, Du M, Deng G, Song Z, Han H (2022). Efficient Gene Silencing in Intact Plant Cells Using siRNA Delivered By Functional Graphene Oxide Nanoparticles. Angew Chem Int Ed Engl.

[53] Conroy F, Miller R, Alterman JF, Hassler MR, Echeverria D, Godinho B (2022). Chemical engineering of therapeutic siRNAs for allele-specific gene silencing in Huntington's disease models. Nat Commun.

[54] Fire A, Xu S, Montgomery MK, Kostas SA, Driver SE, Mello CC (1998). Potent and specific genetic interference by double-stranded RNA in Caenorhabditis elegans. Nature.

[55] Yonezawa S, Koide H, Asai T (2020). Recent advances in siRNA delivery mediated by lipid-based nanoparticles. Adv Drug Deliv Rev.

[56] Scott LJ (2020). Givosiran: First Approval. Drugs.

[57] Liu Y, Ashmawy S, Latta L, Weiss AV, Kiefer AF, Nasr S (2023). pH-Responsive Dynaplexes as Potent Apoptosis Inductors by Intracellular Delivery of Survivin siRNA. Biomacromolecules.

[58] Rossi JJ, Rossi DJ (2021). siRNA Drugs: Here to Stay. Mol Ther.

[59] Gao Y, Chen X, Tian T, Zhang T, Gao S, Zhang X (2022). A Lysosome-Activated Tetrahedral Nanobox for Encapsulated siRNA Delivery. Adv Mater.

[60] Hu B, Zhong L, Weng Y, Peng L, Huang Y, Zhao Y (2020). Therapeutic siRNA: state of the art. Signal Transduct Target Ther.

[61] Deng J, Yang S, Li Y, Tan X, Liu J, Yu Y (2024). Natural evidence of coronaviral 2'-O-methyltransferase activity affecting viral pathogenesis via improved substrate RNA binding. Signal Transduct Target Ther.

[62] Khaitov M, Nikonova A, Shilovskiy I, Kozhikhova K, Kofiadi I, Vishnyakova L (2021). Silencing of SARS-CoV-2 with modified siRNA-peptide dendrimer formulation. Allergy.

[63] Ueda K, Sakagawa Y, Saito T, Sakuma F, Tanaka H, Akita H (2024). NMR-based analysis of impact of siRNA mixing conditions on internal structure of siRNA-loaded LNP. J Control Release.

[64] Ma Z, Wong SW, Forgham H, Esser L, Lai M, Leiske MN (2022). Aerosol delivery of star polymer-siRNA nanoparticles as a therapeutic strategy to inhibit lung tumor growth. Biomaterials.

[65] Badri P, Jiang X, Borodovsky A, Najafian N, Kim J, Clausen VA (2021). Pharmacokinetic and Pharmacodynamic Properties of Cemdisiran, an RNAi Therapeutic Targeting Complement Component 5, in Healthy Subjects and Patients with Paroxysmal Nocturnal Hemoglobinuria. Clin Pharmacokinet.

[66] Pasi KJ, Lissitchkov T, Mamonov V, Mant T, Timofeeva M, Bagot C (2021). Targeting of antithrombin in hemophilia A or B with investigational siRNA therapeutic fitusiran-Results of the phase 1 inhibitor cohort. J Thromb Haemost.

[67] Friedrich M, Aigner A (2022). Therapeutic siRNA: State-of-the-Art and Future Perspectives. BioDrugs.

[68] Halbrook CJ, Lyssiotis CA, Pasca di Magliano M, Maitra A (2023). Pancreatic cancer: Advances and challenges. Cell.

[69] Molitoris BA, Dagher PC, Sandoval RM, Campos SB, Ashush H, Fridman E (2009). siRNA targeted to p53 attenuates ischemic and cisplatin-induced acute kidney injury. J Am Soc Nephrol.

[70] Chen X, Xing X, Lin S, Huang L, He L, Zou Y (2023). Plant-derived nanovesicles: harnessing nature's power for tissue protection and repair. J Nanobiotechnology.

[71] Li Z, Wang H, Yin H, Bennett C, Zhang HG, Guo P (2018). Arrowtail RNA for Ligand Display on Ginger Exosome-like Nanovesicles to Systemic Deliver siRNA for Cancer Suppression. Sci Rep.

[72] Sung J, Yang C, Collins JF, Merlin D (2020). Preparation and Characterization of Ginger Lipid-derived Nanoparticles for Colon-targeted siRNA Delivery. Bio Protoc.

[73] Cui C, Du M, Zhao Y, Tang J, Liu M, Min G (2024). Functional Ginger-Derived Extracellular Vesicles-Coated ZIF-8 Containing TNF-alpha siRNA for Ulcerative Colitis Therapy by Modulating Gut Microbiota. ACS Appl Mater Interfaces.

[74] Rabienezhad Ganji N, Urzi O, Tinnirello V, Costanzo E, Polito G, Palumbo Piccionello A (2023). Proof-of-Concept Study on the Use of Tangerine-Derived Nanovesicles as siRNA Delivery Vehicles toward Colorectal Cancer Cell Line SW480. Int J Mol Sci.

[75] Huang H, Yi X, Wei Q, Li M, Cai X, Lv Y (2023). Edible and cation-free kiwi fruit derived vesicles mediated EGFR-targeted siRNA delivery to inhibit multidrug resistant lung cancer. J Nanobiotechnology.

[76] Itakura S, Shohji A, Amagai S, Kitamura M, Takayama K, Sugibayashi K (2023). Gene knockdown in HaCaT cells by small interfering RNAs entrapped in grapefruit-derived extracellular vesicles using a microfluidic device. Sci Rep.

[77] Xiao J, Tang X, Li Y, Fang Z, Ma D, He Y (2011). Identification of microRNA precursors based on random forest with network-level representation method of stem-loop structure. BMC Bioinformatics.

[78] Diener C, Keller A, Meese E (2022). Emerging concepts of miRNA therapeutics: from cells to clinic. Trends Genet.

[79] Fabian MR, Sonenberg N (2012). The mechanics of miRNA-mediated gene silencing: a look under the hood of miRISC. Nat Struct Mol Biol.

[80] Han J, LaVigne CA, Jones BT, Zhang H, Gillett F, Mendell JT (2020). A ubiquitin ligase mediates target-directed microRNA decay independently of tailing and trimming. Science.

[81] Kleaveland B (2023). SnapShot: Target-directed miRNA degradation. Cell.

[82] Han J, Mendell JT (2023). MicroRNA turnover: a tale of tailing, trimming, and targets. Trends Biochem Sci.

[83] Hong J, Sim D, Lee B-H, Sarangthem V, Park R-W (2024). Multifunctional elastin-like polypeptide nanocarriers for efficient miRNA delivery in cancer therapy. Journal of Nanobiotechnology.

[84] Alden NA, Yeingst TJ, Pfeiffer HM, Celik N, Arrizabalaga JH, Helton AM (2024). Near-Infrared Induced miR-34a Delivery from Nanoparticles in Esophageal Cancer Treatment. Adv Healthc Mater.

[85] Chen X, Chen H, Zhu L, Zeng M, Wang T, Su C (2024). Nanoparticle-Patch System for Localized, Effective, and Sustained miRNA Administration into Infarcted Myocardium to Alleviate Myocardial Ischemia-Reperfusion Injury. ACS Nano.

[86] Pal P, Sharma M, Gupta SK, Potdar MB, Belgamwar AV (2024). miRNA-124 loaded extracellular vesicles encapsulated within hydrogel matrices for combating chemotherapy-induced neurodegeneration. Biochem Biophys Res Commun.

[87] Arora T, Sharma G, Prashar V, Singh R, Sharma A, Changotra H (2024). Mechanistic Evaluation of miRNAs and Their Targeted Genes in the Pathogenesis and Therapeutics of Parkinson's Disease. Mol Neurobiol.

[88] Winkle M, El-Daly SM, Fabbri M, Calin GA (2021). Noncoding RNA therapeutics - challenges and potential solutions. Nat Rev Drug Discov.

[89] Yang Y, Yujiao W, Fang W, Linhui Y, Ziqi G, Zhichen W (2020). The roles of miRNA, lncRNA and circRNA in the development of osteoporosis. Biol Res.

[90] Del Pozo-Acebo L, Lopez de Las Hazas MC, Tome-Carneiro J, Del Saz-Lara A, Gil-Zamorano J, Balaguer L (2022). Therapeutic potential of broccoli-derived extracellular vesicles as nanocarriers of exogenous miRNAs. Pharmacol Res.

[91] Zhu Z, Liao L, Gao M, Liu Q (2023). Garlic-derived exosome-like nanovesicles alleviate dextran sulphate sodium-induced mouse colitis via the TLR4/MyD88/NF-kappaB pathway and gut microbiota modulation. Food Funct.

[92] Zhuang X, Teng Y, Samykutty A, Mu J, Deng Z, Zhang L (2016). Grapefruit-derived Nanovectors Delivering Therapeutic miR17 Through an Intranasal Route Inhibit Brain Tumor Progression. Mol Ther.

[93] Kim J, Zhu Y, Chen S, Wang D, Zhang S, Xia J (2023). Anti-glioma effect of ginseng-derived exosomes-like nanoparticles by active blood-brain-barrier penetration and tumor microenvironment modulation. J Nanobiotechnology.

[94] Corvigno S, Liu Y, Bayraktar E, Stur E, Bayram NN, Ahumada AL (2024). Enhanced plant-derived vesicles for nucleotide delivery for cancer therapy. NPJ Precis Oncol.

[95] Yan G, Xiao Q, Zhao J, Chen H, Xu Y, Tan M (2024). Brucea javanica derived exosome-like nanovesicles deliver miRNAs for cancer therapy. J Control Release.

[96] Yan L, Cao Y, Hou L, Luo T, Li M, Gao S (2024). Ginger exosome-like nanoparticle-derived miRNA therapeutics: A strategic inhibitor of intestinal inflammation. J Adv Res.

[97] Fang E, Liu X, Li M, Zhang Z, Song L, Zhu B (2022). Advances in COVID-19 mRNA vaccine development. Signal Transduct Target Ther.

[98] Szabo GT, Mahiny AJ, Vlatkovic I (2022). COVID-19 mRNA vaccines: Platforms and current developments. Mol Ther.

[99] Tortorici MA, Addetia A, Seo AJ, Brown J, Sprouse K, Logue J (2024). Persistent immune imprinting occurs after vaccination with the COVID-19 XBB.1.5 mRNA booster in humans. Immunity.

[100] Ford ES, Mayer-Blackwell K, Jing L, Laing KJ, Sholukh AM, St Germain R (2024). Repeated mRNA vaccination sequentially boosts SARS-CoV-2-specific CD8(+) T cells in persons with previous COVID-19. Nat Immunol.

[101] Ramos da Silva J, Bitencourt Rodrigues K, Formoso Pelegrin G, Silva Sales N, Muramatsu H, de Oliveira Silva M (2023). Single immunizations of self-amplifying or non-replicating mRNA-LNP vaccines control HPV-associated tumors in mice. Sci Transl Med.

[102] Bevers S, Kooijmans SAA, Van de Velde E, Evers MJW, Seghers S, Gitz-Francois J (2022). mRNA-LNP vaccines tuned for systemic immunization induce strong antitumor immunity by engaging splenic immune cells. Mol Ther.

[103] Pomatto MAC, Gai C, Negro F, Massari L, Deregibus MC, De Rosa FG (2023). Oral Delivery of mRNA Vaccine by Plant-Derived Extracellular Vesicle Carriers. Cells.

[104] Arjmand B, Larijani B, Sheikh Hosseini M, Payab M, Gilany K, Goodarzi P (2020). The Horizon of Gene Therapy in Modern Medicine: Advances and Challenges. Adv Exp Med Biol.

[105] Bucher K, Rodriguez-Bocanegra E, Wissinger B, Strasser T, Clark SJ, Birkenfeld AL (2023). Extra-viral DNA in adeno-associated viral vector preparations induces TLR9-dependent innate immune responses in human plasmacytoid dendritic cells. Sci Rep.

[106] Christie KA, Guo JA, Silverstein RA, Doll RM, Mabuchi M, Stutzman HE (2023). Precise DNA cleavage using CRISPR-SpRYgests. Nat Biotechnol.

[107] Lim M, Badruddoza AZM, Firdous J, Azad M, Mannan A, Al-Hilal TA (2020). Engineered Nanodelivery Systems to Improve DNA Vaccine Technologies. Pharmaceutics.

[108] Wang Q, Zhuang X, Mu J, Deng ZB, Jiang H, Zhang L (2013). Delivery of therapeutic agents by nanoparticles made of grapefruit-derived lipids. Nat Commun.

[109] Hillman T (2023). The use of plant-derived exosome-like nanoparticles as a delivery system of CRISPR/Cas9-based therapeutics for editing long non-coding RNAs in cancer colon cells. Front Oncol.

[110] Zhu Y, Zhu L, Wang X, Jin H (2022). RNA-based therapeutics: an overview and prospectus. Cell Death Dis.

[111] Hammond SM, Abendroth F, Goli L, Stoodley J, Burrell M, Thom G (2022). Antibody-oligonucleotide conjugate achieves CNS delivery in animal models for spinal muscular atrophy. JCI Insight.

[112] Matsuo M (2021). Antisense Oligonucleotide-Mediated Exon-skipping Therapies: Precision Medicine Spreading from Duchenne Muscular Dystrophy. JMA J.

[113] Ye C, Wang M, Min J, Tay RY, Lukas H, Sempionatto JR (2024). A wearable aptamer nanobiosensor for non-invasive female hormone monitoring. Nat Nanotechnol.

[114] Dong Y, Gao Q, Chen Y, Zhang Z, Du Y, Liu Y (2023). Identification of CircRNA signature associated with tumor immune infiltration to predict therapeutic efficacy of immunotherapy. Nat Commun.

[115] Chitkara D, Singh S, Mittal A (2016). Nanocarrier-based co-delivery of small molecules and siRNA/miRNA for treatment of cancer. Ther Deliv.

[116] Gandhi NS, Tekade RK, Chougule MB (2014). Nanocarrier mediated delivery of siRNA/miRNA in combination with chemotherapeutic agents for cancer therapy: current progress and advances. J Control Release.

[117] Pomatto MAC, Gai C, Negro F, Massari L, Deregibus MC, Grange C (2023). Plant-Derived Extracellular Vesicles as a Delivery Platform for RNA-Based Vaccine: Feasibility Study of an Oral and Intranasal SARS-CoV-2 Vaccine. Pharmaceutics.

[118] Li A, Li D, Gu Y, Liu R, Tang X, Zhao Y (2023). Plant-derived nanovesicles: Further exploration of biomedical function and application potential. Acta Pharm Sin B.

[119] Pratiwi FW, Thomas RT, Karzarjeddi M, Sarpola M, Miinalainen I, Makieieva O (2024). Scalable Purification, Storage, and Release of Plant-Derived Nanovesicles for Local Therapy Using Nanostructured All-Cellulose Composite Membranes. Biomacromolecules.

[120] Xu Z, Xu Y, Zhang K, Liu Y, Liang Q, Thakur A (2023). Plant-derived extracellular vesicles (PDEVs) in nanomedicine for human disease and therapeutic modalities. J Nanobiotechnology.

[121] Wang W, He S, Dong G, Sheng C (2022). Nucleic-Acid-Based Targeted Degradation in Drug Discovery. J Med Chem.

[122] Zhang H, Vandesompele J, Braeckmans K, De Smedt SC, Remaut K (2024). Nucleic acid degradation as barrier to gene delivery: a guide to understand and overcome nuclease activity. Chem Soc Rev.

[123] Chen X, Ji S, Yan Y, Lin S, He L, Huang X (2023). Engineered Plant-Derived Nanovesicles Facilitate Tumor Therapy: Natural Bioactivity Plus Drug Controlled Release Platform. Int J Nanomedicine.

